# Predicting the efficiency of chidamide in patients with angioimmunoblastic T-cell lymphoma using machine learning algorithm

**DOI:** 10.3389/fphar.2024.1435284

**Published:** 2024-08-28

**Authors:** Chunlan Zhang, Juan Xu, Mingyu Gu, Yun Tang, Wenjiao Tang, Jie Wang, Qinyu Liu, Yunfan Yang, Xushu Zhong, Caigang Xu

**Affiliations:** ^1^ Department of Hematology, Institute of Hematology, West China Hospital, Sichuan University, Chengdu, China; ^2^ West China School of Medicine and West China Hospital, Sichuan University, Chengdu, China

**Keywords:** machine learning, chidamide, prognosis, angioimmunoblastic T-cell lymphoma, biomarker

## Abstract

**Background:**

Chidamide is subtype-selective histone deacetylase (HDAC) inhibitor that showed promising result in clinical trials to improve prognosis of angioimmunoblastic T-cell lymphoma (AITL) patients. However, in real world settings, contradictory reports existed as to whether chidamide improve overall survival (OS). Therefore, we aimed to develop an interpretable machine learning (Machine learning)–based model to predict the 2-year overall survival of AITL patients based on chidamide usage and baseline features.

**Methods:**

A total of 183 patients with AITL were randomly divided into training set and testing set. We used 5 ML algorithms to build predictive models. Recursive feature elimination (RFE) method was used to filter for the most important features. The ML models were interpreted and the relevance of the selected features was determined using the Shapley additive explanations (SHAP) method and the local interpretable model–agnostic explanationalgorithm.

**Results:**

A total of 183 patients with newly diagnosed AITL from 2012 to 2022 from 3 centers in China were enrolled in our study. Seventy-one patients were dead within 2 years after diagnosis. Five ML algorithms were built based on chidamide usage and 16 baseline features to predict 2-year OS. Catboost model presented to be the best predictive model. After RFE screening, 12 variables demonstrated the best performance (AUC = 0.8651). Using chidamide ranked third among all the variables that correlated with 2-year OS.

**Conclusion:**

This study demonstrated that the Catboost model with 12 variables could effectively predict the 2-year OS of AITL patients. Combining chidamide in the treatment therapy was positively correlated with longer OS of AITL patients.

## Introduction

Angioimmunoblastic T-cell lymphoma (AITL) is a distinct kind of peripheral T-cell lymphoma (PTCL) that has a poor prognosis (Swerdlow et al., 2016). For AITL patients, the 5-year overall survival (OS) rate was 44% and the progression-free survival (PFS) rate was 32% (Advani et al., 2021). Anthracycline-based chemotherapy regimens are frequently utilized, yet their effectiveness is constrained. Based on the unsatisfactory outcome of traditional treatment, the NCCN Clinical Practice Guidelines in Oncology recommended engaging in clinical trials as preferred management strategy (Horwitz et al., 2022). Notably, although some patients had the identical staging or prognostic scores that commonly used to evaluate T cell lymphoma, their clinical outcomes varied considerably. The differences in prognosis may be due to the heterogeneity of AITL (Zhang et al., 2023). Therefore, novel models that can better stratify patients are required.

Chidamide is a benzamide type of subtype-selective histone deacetylase (HDAC) inhibitor (Gong et al., 2012). In recent years, chidamide has appeared as a promising treatment in PTCL, especially in AITL. In phase II study of chidamide in relapsed or refractory (r/r) AITL, the overall response rate (ORR) was 50% (Shi et al., 2015). In a multicenter phase II clinical trial combining chidamide with prednisone, etoposide, and thalidomide in untreated AITL, the ORR was 90.2%. The 2-year progression-free survival (PFS) rate and overall survival (OS) rate were 66.5% and 82.2%, respectively (Wang et al., 2022b). However, in real world analysis, contradictory results exist as for whether combining chidamide with chemotherapy improves OS compared with chemotherapy alone (Shi et al., 2017; Liu et al., 2021; Wang et al., 2022a). Further evidence is required to clarify the efficiency of chidamide in real-world setting.

Machine learning (ML) algorithms is a key area of artificial intelligence, which may learn from complicated data by utilizing computational methods to identify possible features for prediction (Haug and Drazen, 2023). Compared with conventional generalized linear model, machine learning based on the advanced algorithm are more acceptable in terms of data distribution and integrity, as well as more flexible in terms of mining data value (Elemento et al., 2021). Therefore, machine learning has been widely used in the medical field in recent years and has developed into a potent tool for physicians to use when making clinical decisions (Radakovich et al., 2020; Haug and Drazen, 2023; Swanson et al., 2023). Hence, the purposes of the present study were to establish ML models to predict prognosis of AITL and to evaluate the benefit of chidamide in real-world setting.

## Materials and methods

### Patients

This retrospective multicenter study included patients with newly diagnosed AITL between 2012 and 2022 from three centers in China, and this study was approved by the Ethics Committees of three hospitals.

### Data collection

Data on the clinical and laboratory metrics of patients were obtained from three hospital databases. The inclusion criteria were strictly adhered to, ensuring that all participants: (1) had a confirmed pathological diagnosis of AITL, (2) newly diagnosed cases, (3) were aged over 18 years, and (4) were capable of independently signing informed consent. Baseline information includes a total of 16 variables: (1) basic condition: age, gender; (2) the condition of lymphoma: Ann Arbor staging, B symptom, extranodal involvement, Eastern Cooperative Oncology Group (ECOG) Performance Status, International Prognostic Index (IPI); (3) clinical symptoms: rash, edema/serous effusion; (4) laboratory parameters: hemoglobin (Hb), platelet (PLT), absolute lymphocyte count (ALC), absolute eosinophil count (AEC), serum albumin (ALB), serum globulin (GLB), lactate dehydrogenase (LDH).

Patients were treated with chemotherapy, mostly anthracycline-based regimens, with or without chidamide. We conducted patient follow-up monthly and recorded the detailed treatment strategy and clinical outcomes. Participants with significant data omissions were excluded to maintain the integrity and reliability of our research.

### Study design and machine learning algorithms

A total of 17 variables were included to identify the prognostic value of these features, including 16 baseline features and “chidamide usage”. Among 183 patients, 71 patients died within 2 years after diagnosis, while 112 patients were still alive. About 75% (137) of patients were randomly selected into the training set, while the rest 46 patients fell into the validation set. Five models were built through logistics regression (LR), random forest (RF), light gradient boosting machine (LGBM), extreme gradient boosting (XGBoost), and categorical boosting (CatBoost) to predict 2-year OS. The selection of models was based on a comprehensive consideration of performance, interpretability, computational efficiency, generalizability, as well as support by literature documenting similar research contexts, ensuring the relevance and robustness of our approach.

### Model validation

We explored five different machine learning algorithms and conducted grid search techniques to identify the best hyperparameter combinations, setting possible value ranges for different parameters and evaluating each parameter combination with 5-fold cross-validation. The receiver-operating characteristic (ROC) curve was used as the assessment metric to validate the performances of the different models. The performance of each model was evaluated by calculating the area under the curve (AUC), accuracy, precision, recall, specificity, and F1 score. After comparing the AUC values of the five algorithms, we chose CatBoost as our final model due to its superior performance and high AUC value on the test set. The parameters for five models were provided in Supplementary Table 1.

We implemented Recursive Feature Elimination (RFE) with the CatBoost algorithm to reduce the number of features and enhance model efficiency. RFE is a model-based feature selection method that methodically reduces the feature set by recursively removing the least significant feature during each iteration. By meticulously comparing the performance across different feature subsets, we selected 12 features that contribute most significantly to the model’s predictive power. The model demonstrated good performance and robustness on the test set, highlighting its generalizability.

### Model interpretation

The interpretation of the predictions produced by the models was conducted using the Shapley additive explanations (SHAP) value. The SHAP technique is capable of providing a more comprehensive explanation of the significance of each variable in all component sequences by doing a marginal calculation of their contributions. The predictions were also explained using the local interpretable model–agnostic explanation (LIME). The trustworthiness of a model’s explanation for predicting a single sample using a local linear approximation of the model’s behavior can be enhanced.

### Statistical analysis

Patients were categorized into two groups based on their 2-year status: alive and dead. Binary variables were subjected to either a Fisher exact test or a *γ* ^2^ test. For continuous variables that adhere to the normal distribution, a Student’s t-test was employed, and the data were displayed as medians along with standard deviations. For variables that do not follow the normal distribution, a Mann-Whitney *U* test was utilized, and the data were presented as medians along with interquartile ranges (IQRs). The development and validation of the five machine learning algorithms were conducted using Python software. Statistical significance was set at *p* ≤ 0.05. Python software (version 3.9.13) was utilized to perform statistical analysis.

## Results

### Patient characteristics

**FIGURE 1:**
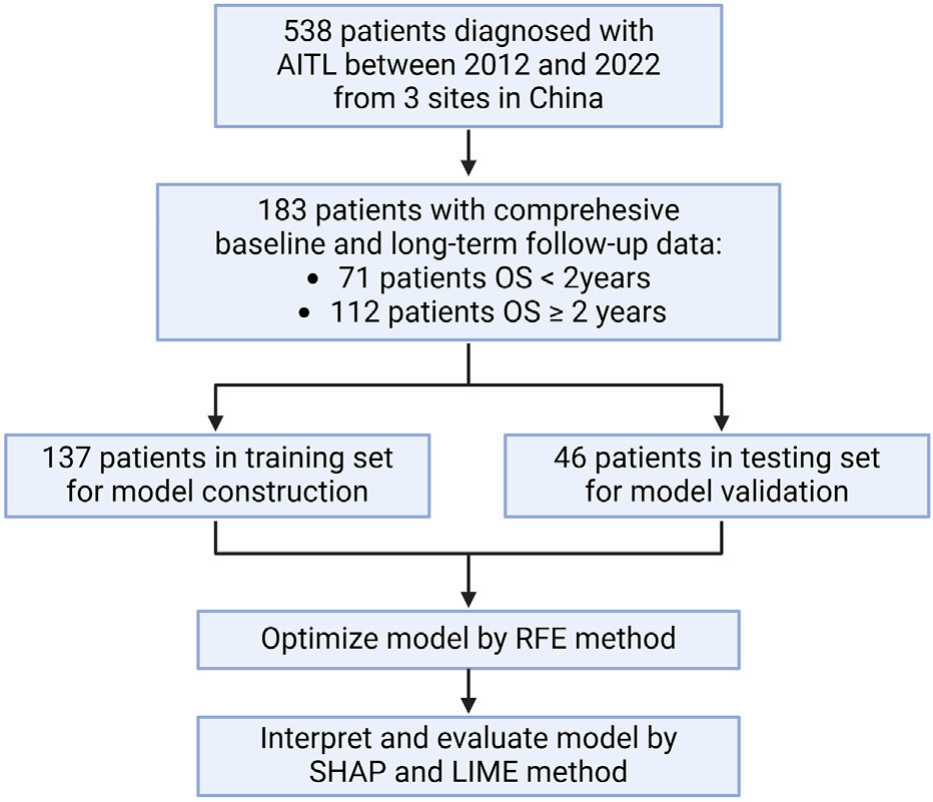
Flowchart. OS: overall survival; RFE: recursive feature elimination; SHAP: Shapley additive explanations; LIME, local interpretable model–agnostic explanation.

A total of 538 patients were newly diagnosed with AITL between 2012 and 2022 from three centers in China. Following the screening process, we included 183 patients who had a comprehensive baseline examination and long-term follow-up data of at least 24 months in our study (Figure 1). Cohort characteristics are presented in Table 1. The median age was 63 (IQR = 54–68) years and 63.39% were males. AITL patients were categorized into two groups based on 2-year OS. Among 183 patients, 112 patients had an OS longer than 2 years.

**TABLE 1:** Clinical and laboratory data of AITL patients.

Parameter	Total (n = 183)	Alive (n = 112)	Dead (n = 71)	*p*-value
Gender				0.876
Male	116 (63.39%)	70 (62.5%)	46 (64.79%)	
Female	67 (36.61%)	42 (37.5%)	25 (35.21%)	
Age	63.0 (54.0, 68.0)	62.0 (54.0, 68.0)	65.0 (54.0, 70.0)	0.135
Edema/Serous effusion				<0.001**
Yes	97 (53.01%)	44 (39.29%)	53 (74.65%)	
No	86 (46.99%)	68 (60.71%)	18 (25.35%)	
Rash				0.995
Yes	55 (30.05%)	34 (30.36%)	21 (29.58%)	
No	128 (69.95%)	78 (69.64%)	50 (70.42%)	
IPI				<0.001**
0–1	36 (19.67%)	27 (24.11%)	9 (12.68%)	
2	40 (21.86%)	27 (24.11%)	13 (18.31%)	
3	69 (37.7%)	46 (41.07%)	23 (32.39%)	
4–5	38 (20.77%)	12 (10.71%)	26 (36.62%)	
ECOG				<0.001**
0	76 (41.53%)	56 (50.0%)	20 (28.17%)	
1	63 (34.43%)	40 (35.71%)	23 (32.39%)	
2–4	44 (24.04%)	16 (14.29%)	28 (39.44%)	
Extranodal involvement				0.048*
Yes	80 (43.72%)	42 (37.5%)	38 (53.52%)	
No	103 (56.28%)	70 (62.5%)	33 (46.48%)	
B symptom				<0.001**
Yes	104 (56.83%)	51 (45.54%)	53 (74.65%)	
No	79 (43.17%)	61 (54.46%)	18 (25.35%)	
Ann Arbor staging				0.036*
1–2	19 (10.38%)	16 (14.29%)	3 (4.23%)	
3	90 (49.18%)	57 (50.89%)	33 (46.48%)	
4	74 (40.44%)	39 (34.82%)	35 (49.3%)	
Hb	118.0 (95.0, 130.0)	119.0 (103.75, 131.0)	117.0 (90.0, 127.0)	0.026*
PLT	160.0 (127.0, 212.5)	160.0 (138.25, 213.0)	157.0 (104.52, 212.5)	0.148
ALC	0.93 (0.63, 1.33)	0.955 (0.708, 1.435)	0.87 (0.595, 1.095)	0.031*
AEC	0.13 (0.06, 0.24)	0.13 (0.06, 0.22)	0.13 (0.06, 0.31)	0.576
ALB	37.0 (31.9, 40.5)	38.0 (33.8, 41.85)	34.4 (29.45, 38.1)	<0.001**
GLB	29.5 (25.2, 36.7)	29.25 (24.82, 35.33)	30.2 (26.0, 38.25)	0.172
LDH	254.0 (192.5, 329.0)	236.5 (187.75, 310.0)	296.0 (215.0, 390.5)	0.013*
Chidamide				0.064
Yes	112 (61.2%)	75 (66.96%)	37 (52.11%)	
No	71 (38.8%)	37 (33.04%)	34 (47.89%)	

ECOG, Eastern Cooperative Oncology Group performance status; IPI, International Prognostic Index; Hb, hemoglobin; PLT, platelet; ALC, absolute lymphocyte count; AEC, absolute eosinophil count; ALB, serum albumin; GLB, serum globulin; LDH, lactate dehydrogenase. * *p*-value < 0.05. ** *p*-value < 0.01.

### Predicting 2-year OS by baseline features

Our modeling utilized a comprehensive set of 17 variables, encompassing age, gender, Ann Arbor staging, B symptom, extranidal involvement, ECOG, IPI, rash, edema/serous effusion, Hb, PLT, ALC, AEC, ALB, GLB, LDH, and chidamide. The patients were divided into two groups, namely, the training set for model development and the testing set for model performance evaluation, using a random stratification method with a ratio of 3:1. The clinical characteristics of the two data sets are presented in Table 2. The *p*-values of all features in the training set and the test set were greater than 0.05, indicating that the division of the training and test sets is reasonable.

**TABLE 2:** Clinical and laboratory data of AITL patients categorized by training set and testing set.

Parameter	Training set (n = 137)	Testing set (n = 46)	*p*-value
Gender			0.904
Male	86 (62.77%)	30 (65.22%)	
Female	51 (37.23%)	16 (34.78%)	
Age	62.0 (53.0, 68.0)	63.5 (57.25, 69.75)	0.284
Edema/Serous effusion			0.47
Yes	70 (51.09%)	27 (58.7%)	
No	67 (48.91%)	19 (41.3%)	
Rash			0.534
Yes	39 (28.47%)	16 (34.78%)	
No	98 (71.53%)	30 (65.22%)	
IPI			0.842
0–1	28 (20.44%)	8 (17.39%)	
2	29 (21.17%)	11 (23.91%)	
3	50 (36.5%)	19 (41.3%)	
4–5	30 (21.9%)	8 (17.39%)	
ECOG			0.944
0	56 (40.88%)	20 (43.48%)	
1	48 (35.04%)	15 (32.61%)	
2–4	33 (24.09%)	11 (23.91%)	
Extranodal involvement			0.893
Yes	59 (43.07%)	21 (45.65%)	
No	78 (56.93%)	25 (54.35%)	
B symptom			0.052
Yes	84 (61.31%)	20 (43.48%)	
No	53 (38.69%)	26 (56.52%)	
Ann Arbor staging			0.746
1–2	13 (9.49%)	6 (13.04%)	
3	69 (50.36%)	21 (45.65%)	
4	55 (40.15%)	19 (41.3%)	
Hb	118.0 (95.0, 130.0)	118.5 (96.0, 133.0)	0.665
PLT	160.0 (127.0, 213.0)	156.5 (127.5, 203.0)	0.683
ALC	0.93 (0.62, 1.37)	0.9 (0.65, 1.23)	0.895
AEC	0.13 (0.06, 0.23)	0.12 (0.06, 0.28)	0.744
ALB	37.0 (32.0, 40.5)	36.6 (31.82, 40.45)	0.872
GLB	29.6 (25.0, 36.8)	28.0 (25.42, 35.93)	0.384
LDH	254.0 (199.0, 332.0)	239.5 (188.25, 320.0)	0.673
Chidamide			0.819
Yes	85 (62.04%)	27 (58.7%)	
No	52 (37.96%)	19 (41.3%)	

ECOG, Eastern Cooperative Oncology Group performance status; IPI, International Prognostic Index; Hb, hemoglobin; PLT, platelet; ALC, absolute lymphocyte count; AEC, absolute eosinophil count; ALB, serum albumin; GLB, serum globulin; LDH, lactate dehydrogenase.

**FIGURE 2:**
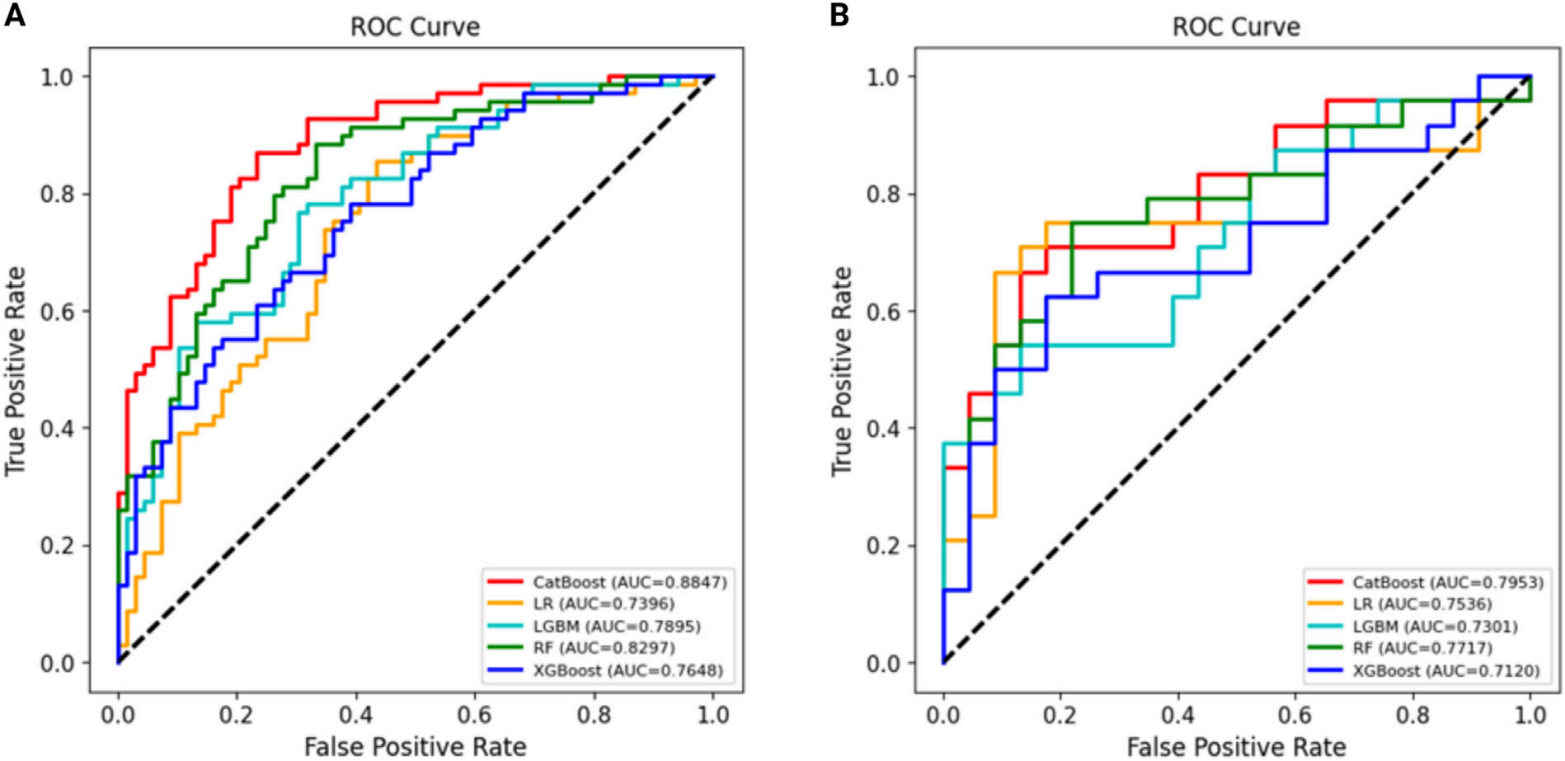
The ROC curves of five ML models. [**(A)** Training set. **(B)** Testing set].

Using all 17 variables, predictive models for 2-year survival were developed based on five algorithms, including LR, RF, LGBM, XGBoost, and CatBoost. The best predictive performance was observed in CatBoost (training set AUC = 0.8949, testing set AUC = 0.8571). The AUC, accuracy, precision, recall, specificity, and F1 score of five models in the training and testing data sets were shown in Table 3. The ROC curves of five models were presented in Figure 2.

**TABLE 3:** Predictive performance of 5 ML models.

Model	AUC (training set)	AUC (testing set)	Accuracy (training set)		Precision (training set)	Recall (training set)	Specificity (training set)	F1
LR	0.7311	0.7937	0.8043	0.7647	0.7222	0.8571	0.7429	0.7311
Random Forest	0.8794	0.8353	0.8043	0.7143	0.8333	0.7857	0.7692	0.8794
LGBM	0.8704	0.8393	0.7826	0.6667	0.8889	0.7143	0.7619	0.8704
XGBoost	0.8971	0.8294	0.8043	0.8462	0.6111	0.9286	0.7097	0.8971
CatBoost	0.8949	0.8571	0.8261	0.7273	0.8889	0.7857	0.8000	0.8949

**FIGURE 3:**
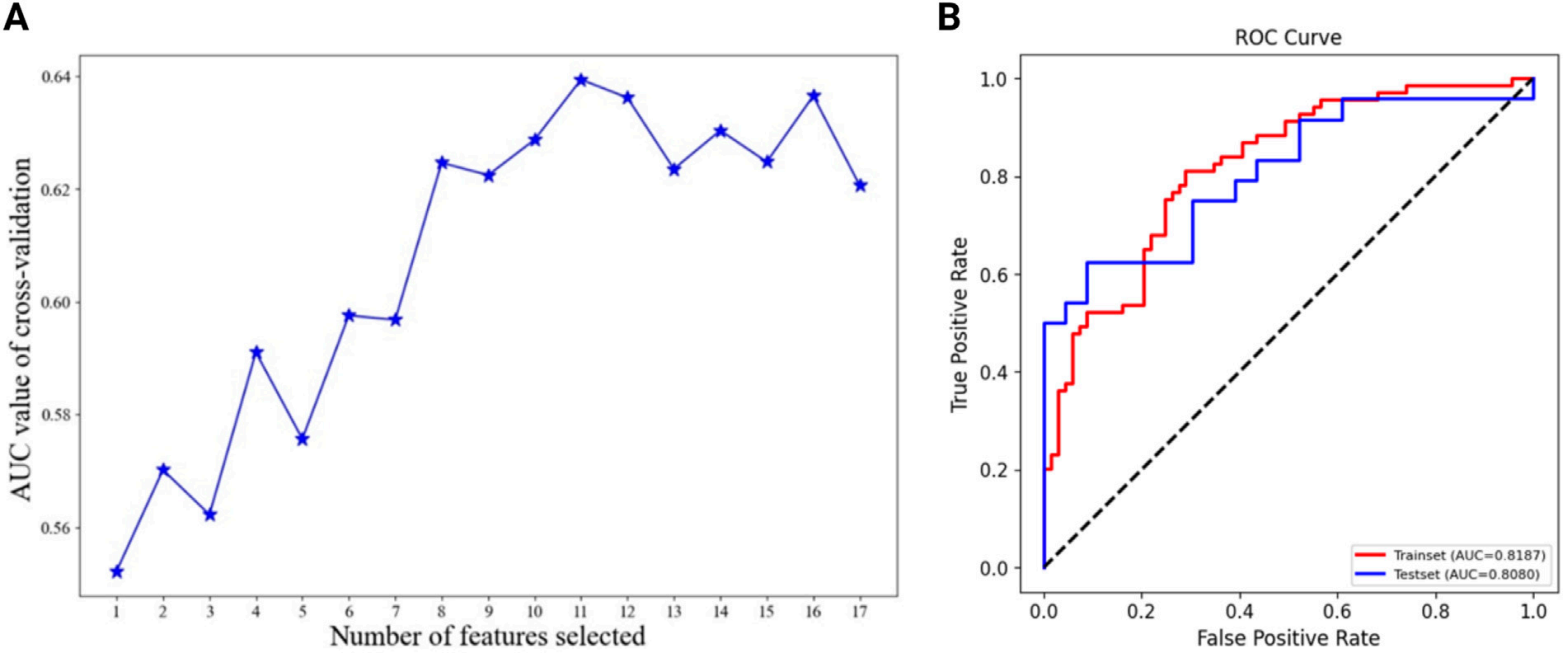
Using RFE method to screen the optimal variables on Catboost model **(A)**. The ROC curves of the optimized Catboost model **(B)**.

The Catboost model was further optimized by employing the recursive feature elimination (RFE) method to selectively filter for the most significant characteristics. The feature importance ranking by RFE was presented in Supplementary Figure 1. In the ideal Catboost algorithm, a total of 12 factors were utilized. These variables encompassed Age, B symptom, Extranodal involvement, ECOG, IPI, Edema/Serous effusion, ALC, AEC, ALB, GLB, LDH, and Chidamide (Figure 3). Table 4 showed the AUC, accuracy, precision, recall, specificity, and F1 score of the optimized Catboost model.

**TABLE 4:** Predictive performance of the optimized Catboost model.

Model	AUC (training set)	AUC (testing set)	Accuracy (training set)	Precision (training set)	Recall (training set)	Specificity (training set)	F1
CatBoost	0.8801	0.8651	0.8478	0.7619	0.8889	0.8214	0.8205

### Interpretation and evaluation of machine learning model

**FIGURE 4:**
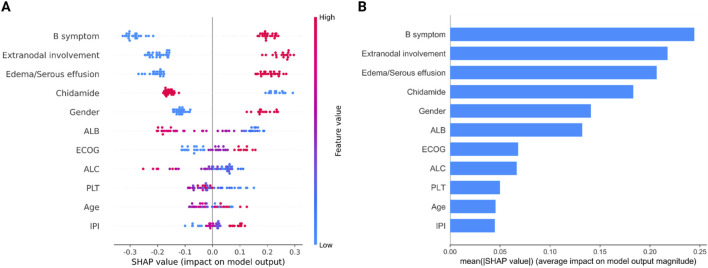
Attribution of 12 features in the optimized Catboost model based on the SHAP algorithm. **(A)** Summary of SHAP analysis on the data set. One dot represents a case in the data set, and the color of a dot indicates the value of the feature. Blue indicates the lowest range and red the highest range. **(B)** Ranking of feature importance indicated by SHAP. SHAP values provide a clear depiction of how each feature influences the model’s prediction, indicating whether the impact is positive or negative. For example, from the SHAP plot, we can observe that B symptoms have the most significant contribution. A higher value of B symptoms corresponds to a positive SHAP value, suggesting a positive impact on the prediction, supporting a likelihood of death. Conversely, a lower value of B symptoms results in a negative SHAP value, indicating a negative impact on the prediction, supporting survival.

We employed the SHAP approach to quantitatively assess the impact of each feature on the prediction outcomes of the model (Figure 4). The analysis of feature ranking revealed that the five most significant features were B symptom, Edema/Serous effusion, Chidamide, Extranodal involvement, and ECOG. Chidamide usage was negatively correlated with the outcome (OS shorter than 2 years), indicating the efficiency of chidamide on improving OS in AITL patients.

**FIGURE 5:**
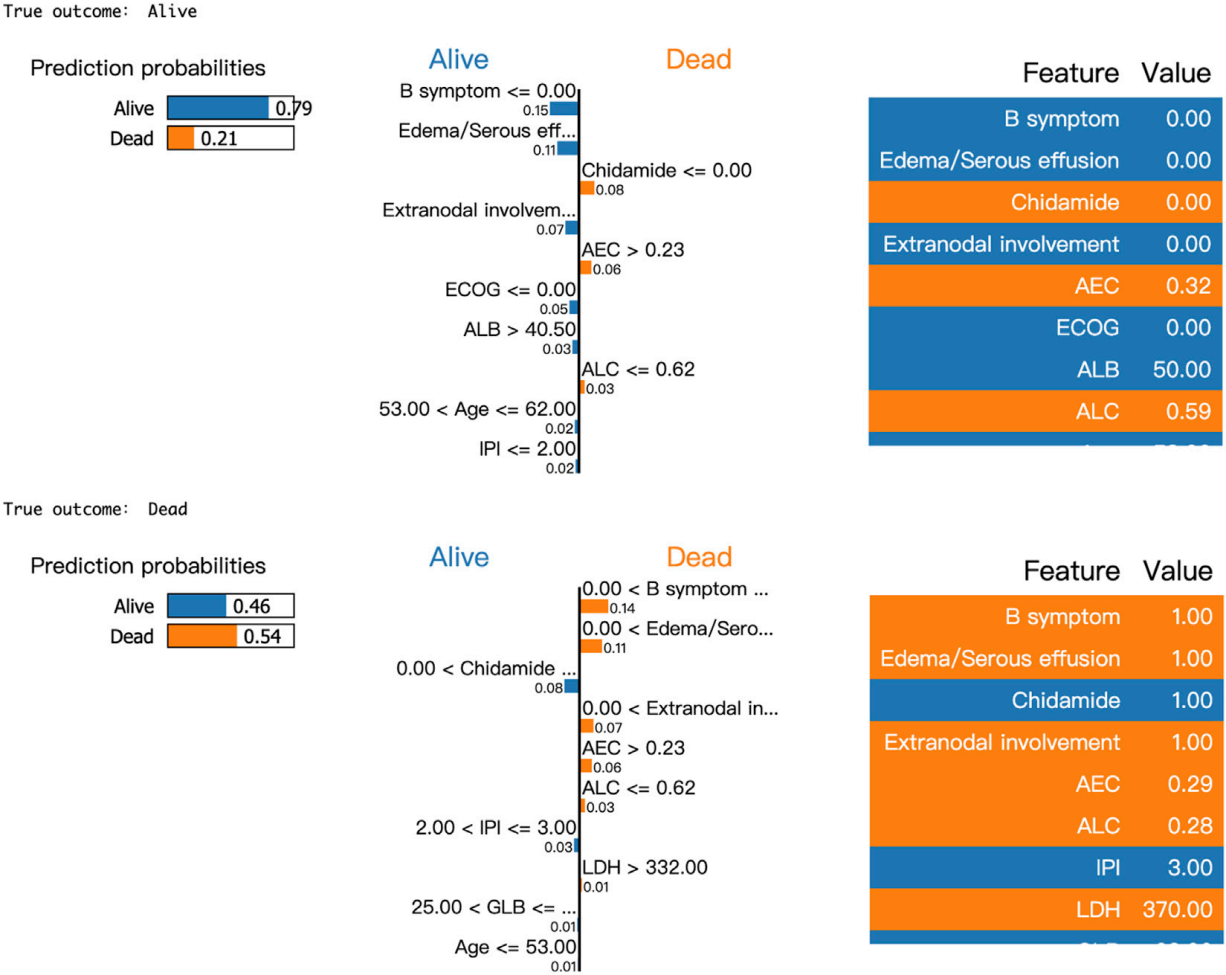
Results of LIME with Catboost applied to two randomly selected patients. Orange attributes support death, while blue attributes support survival. Taking the first patient as an example, the upper left corner of the LIME plot shows a survival prediction probability of 0.79 and a death prediction probability of 0.21, indicating a higher likelihood of survival for the patient within 2 years. The middle section lists the individual features along with their corresponding coefficients, which reflect their impact on the prediction. For instance, the coefficient for B symptoms is 0.14, shown in blue, suggesting that the absence of B symptoms increases the model’s predicted probability of survival by 0.14. Similarly, the absence of Edema/Serous effusion increases the survival probability by 0.11, while the absence of Chidamide use increases the probability of death by 0.08.

Moreover, LIME algorithm was applied to explain the influence of different variables of the Catboost model on the prediction results. Two cases were randomly selected to interpret the visualized prediction results (Figure 5).

## Discussion

AITL is a life-threatening lymphoma with heterogeneous nature. Current staging systems are far from satisfactory to stratify AITL patients. Novel models that can better stratify patients are required. By establishing Catboost model, our study demonstrated that 12 features, including Age, B symptom, Extranodal involvement, ECOG, IPI, Edema/Serous effusion, ALC, AEC, ALB, GLB, LDH, and Chidamide, were significantly related with 2-year OS of AITL patients.

Chidamide, a HDAC inhibitor, has presented as a promising target therapy in recent years in PTCL patients. In clinical trials, chidamide remarkably prolonged the OS of AITL patients. However, in real world analysis, contradictory results exist as for whether combining chidamide with chemotherapy improves OS compared with chemotherapy alone (Shi et al., 2017; Liu et al., 2021; Wang et al., 2022a). Further evidence is required to clarify the efficiency of chidamide in real-world setting. Using ML algorithm, we demonstrated that using chidamide was among the most important features that influent 2-year OS. Specially, in the optimized Catboost model, chidamide ranked third among all the variables that correlated with OS. In general, our study supported that in real-world setting, combining chidamide in treatment strategy could prolong OS of AITL patients.

Chidamide was administered as the initial treatment for 68 patients, as the second line of treatment for 22 patients, and as the maintenance treatment for 43 patients. For patients treated with chemotherapy without Chidamide, no other substitute was added. Nevertheless, the subset analysis revealed that the disparity in response rates between chemotherapy combined with chidamide and without chidamide was not statistically significant in both first line and second line treatment, owing to the small sample size. Out of the 43 patients who received chidamide as a form of maintenance therapy, 24 patients had available treatment responses both before and after the maintenance period. The treatment responses in the majority of patients (22 cases) were consistent both before and after maintenance. It is worth mentioning that two patients demonstrated improved treatment response following the administration of chidamide. There was no evidence of any progressive disease. Previously, the benefit of chidamide as maintenance therapy in real world setting was also reported by Guo et al. (2022).

Similar to the commonly used prognostic scores in T-cell lymphoma, including International Prognostic Index (IPI), Prognostic Index for T-cell lymphoma (PIT), International peripheral T-cell lymphoma Project score (IPTCLP) and modified Prognostic Index for T-cell lymphoma (mPIT), age, B symptom, Extranodal involvement, ECOG, PLT, and LDH were correlated with OS in our study (Gutiérrez-García et al., 2011). Moreover, ALC was also significantly related with 2-year OS, which was in agreement with the AITL score established based on 282 patients with AITL enrolled between 2006 and 2018 in the international prospective T-cell Project (Advani et al., 2021). Interestingly, we also showed that AEC was associated with prognosis, which has not been reported before.

Remarkably, unlike aforementioned prognostic scores, we showed that Edema/Serous effusion and ALB level were also significantly correlated with prognosis. In the optimized Catboost model, Edema/Serous effusion was found to be the second most significant variable. Previously, Sun et al. reported the correlation of serous effusion with OS based on a cohort of 55 AITL patients (Sun et al., 2021), while Huang et al. reported that ALB <30 g/L was significantly associated with poor prognosis base on a cohort of 64 AITL patients (Huang et al., 2020). From a clinical perspective, there was a correlation between Edema/Serous effusion and a reduction in ALB levels. This pair of features warrant more attention in further studies of AITL.

The current investigation possesses several limitations that should not be ignored. First, this study was conducted retrospectively and had a limited sample size. Additional validation in large cohorts is required to further substantiate the efficacy of the model. Second, present study identified chidamide as an effective treatment to AITL patients that prolonged their OS. Nevertheless, the treatment regimens employing chidamide exhibited variability among patients in this retrospective investigation, encompassing factors such as the timing and duration of therapy. Hence, it is imperative to conduct additional validation of the efficacy of chidamide in a substantial prospective cohort within a real-world context. Furthermore, the absence of complete data has resulted in the exclusion of certain potential influencing elements, such as C-reactive protein and Beta-2-microglobulin, from the establishment of machine learning models. Moreover, along with the development of genome and transcriptome analysis, molecular abnormalities which may impact the prognosis of lymphoma were gradually unveiled (Cortes et al., 2018; Lone et al., 2021; Leca et al., 2023; Zhang et al., 2024). In the age of precision medicine, as sequencing technology becomes increasingly prevalent, it is imperative to incorporate these molecular abnormalities into future prognostic models of AITL.

## Conclusion

The Catboost model developed in our study shown a strong prediction capability for the 2-year OS of patients with AITL. Chidamide, in particular, was one of the most significant factors that influenced OS. The utilization of chidamide exhibited a strong correlation with an improved outcome. Further validation of the model’s predictive value should be conducted using larger cohorts.

## Data Availability

The original contributions presented in the study are included in the article/Supplementary Material, further inquiries can be directed to the corresponding author.
