# Supplementary material for: Predicting the efficiency of chidamide in patients with angioimmunoblastic T-cell lymphoma using machine learning algorithm
Source: Front Pharmacol. 2024 Aug 28;15:1435284. doi: 10.3389/fphar.2024.1435284 (PMC11387163; doi:10.3389/fphar.2024.1435284)

**Supplementary Table 1. The detailed parameters of the 5 Algorithms.**

| Algorithms | Parameters |
| --- | --- |
| Logistics Regression | class_weight={0:0.1, 1:0.9}, penalty='l1', solver='liblinear', random_state=2024 |
| Random Forest | n_estimators=100, max_depth=3, min_samples_split=10, min_samples_leaf=4, max_features=1, oob_score=True, random_state=2023 |
| LGBM | boosting_type='gbdt' ,objective='binary' ,learning_rate=0.02 ,n_estimators=100 ,max_depth=4 ,num_leaves=30 ,min_child_samples=6 ,min_child_weight=1 ,bagging_fraction=1 ,feature_fraction=0.9 ,subsample=1 ,colsample_bytree=0.8 ,reg_alpha=5 ,reg_lambda=10 ,random_state=2024 |
| XGBoost | objective='binary:logistic' ,learning_rate=0.02 ,n_estimators=230 ,max_depth=4 ,min_child_weight=5 ,gamma=0 ,subsample=0.8 ,colsample_bytree=1 ,scale_pos_weight=1 ,reg_alpha=2 ,reg_lambda=1 ,n_jobs=-1, eval_metric=['logloss','auc','error'], seed=2024 |
| CatBoost | loss_function="Logloss", eval_metric="AUC", learning_rate=0.01, iterations=1000, random_seed=42, od_type="Iter", depth=4, early_stopping_rounds=800, colsample_bylevel=0.1, l2_leaf_reg=40, random_strength=800, scale_pos_weight=1, silent=True |

**Supplementary Figure 1. Feature importance ranking by RFE.**


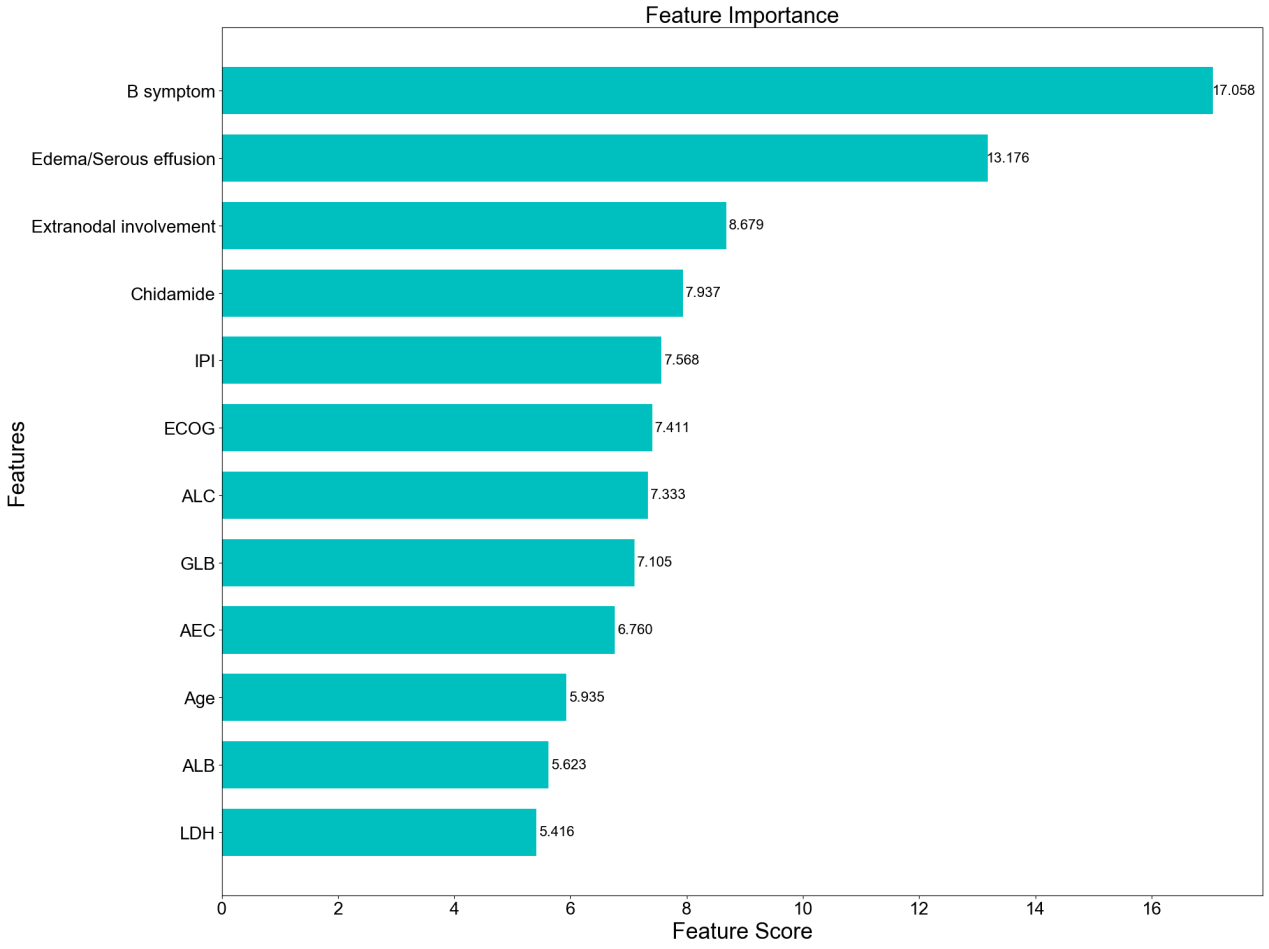

Supplement: Supplementary file 1 [file DataSheet1.DOCX]
